# Supplementary material for: Study of Consumer Liking of Six Chinese Vinegar Products and the Correlation between These Likings and the Volatile Profile
Source: Foods. 2022 Jul 26;11(15):2224. doi: 10.3390/foods11152224 (PMC9332478; doi:10.3390/foods11152224)
Supplement: Supplementary file 1 [file foods-11-02224-s001.zip › foods-1813812-supplementary.pdf]

Supplementary material

Consumer preference of six Chinese vinegar products and the correlation between these preferences and the volatile profile

Contents:

Figure S1. GC–MS total ion chromatograms of Ten-year aged Qian-he cellar vinegar (a), Ning-hua-mansion old vinegar (b), East-lake health vinegar (c), Qian-he glutinous rice vinegar (d), Heng-shun Jinyou balsamic vinegar (e), and potato vinegar (f)

Figure S2. Geographical distribution of respondents

Table S1. Coefficient of PLSR models for each cluster of consumers

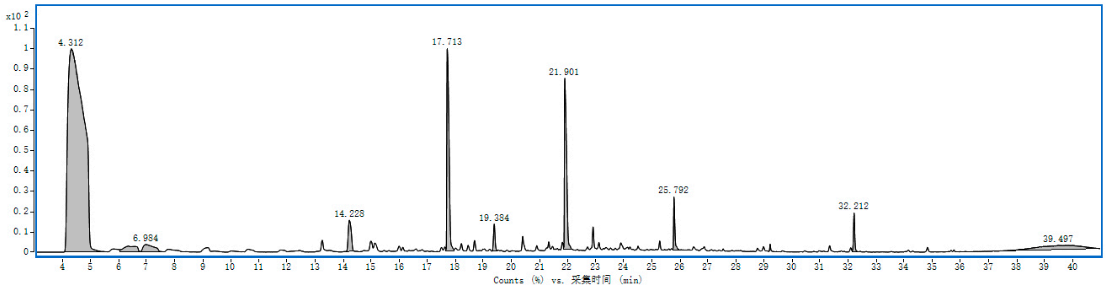
x10 2
4.312
6.984
14.228
17.713
19.384
21.901
25.792
32.212
39.497
Counts (%) vs. 采集时间 (min)

（a）

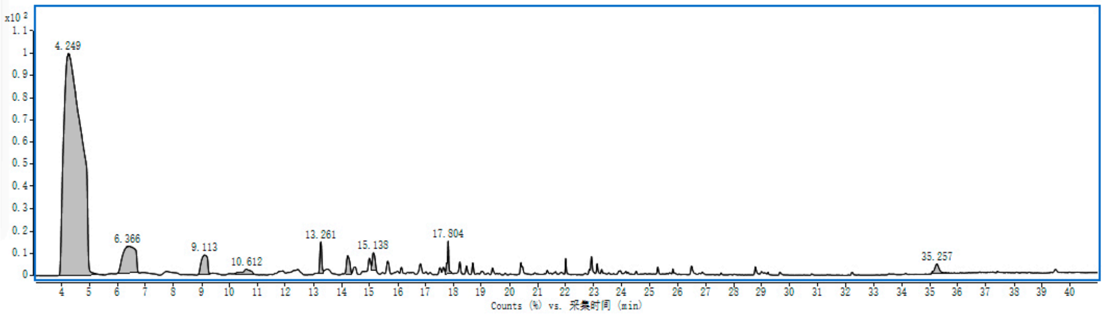
x10 2
4.249
6.366
9.113
10.612
13.261
15.138
17.804
35.257
Counts (%) vs. 采集时间 (min)

（b）

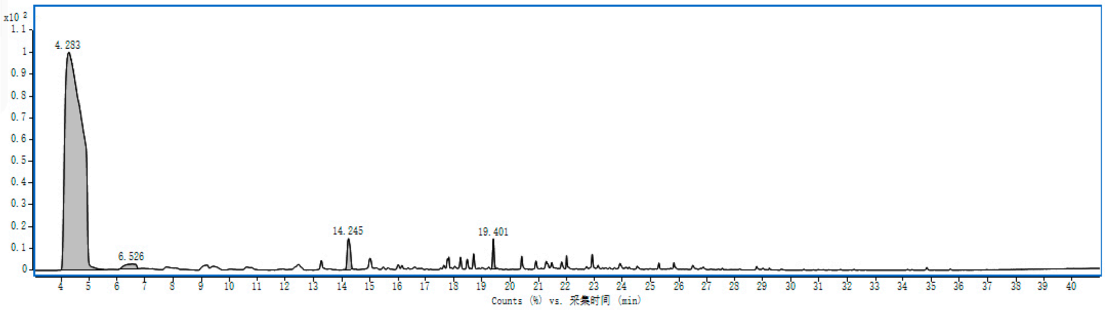
x10 2
4.283
6.526
14.245
19.401
Counts (%) vs. 采集时间 (min)

（c）

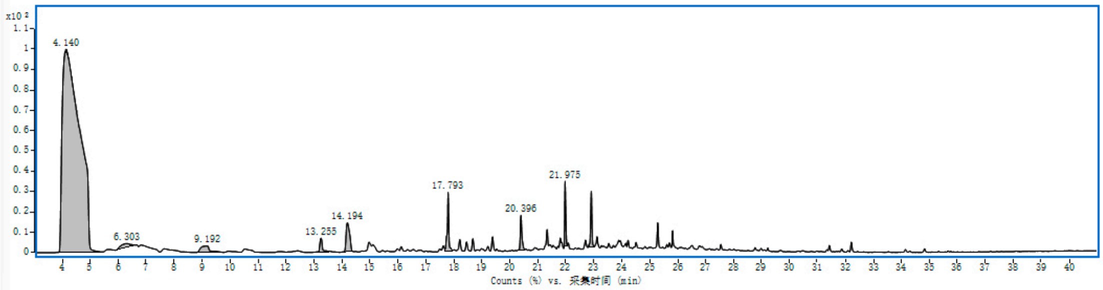
x10 2
4.140
6.303
9.192
13.255
14.194
17.793
20.396
21.975
Counts (%) vs. 采集时间 (min)

（d）

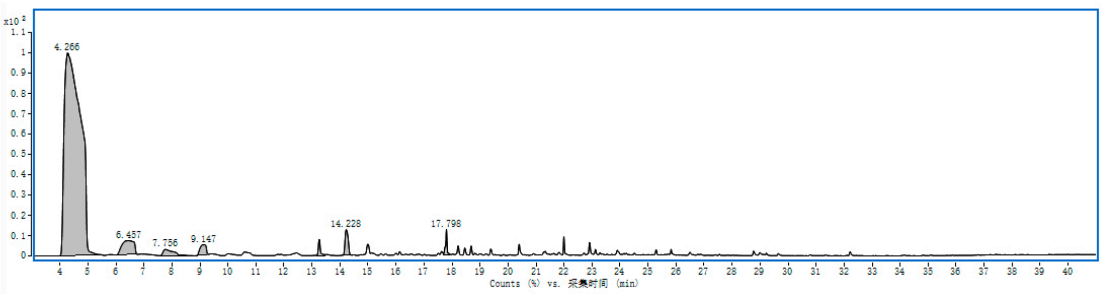

（e）

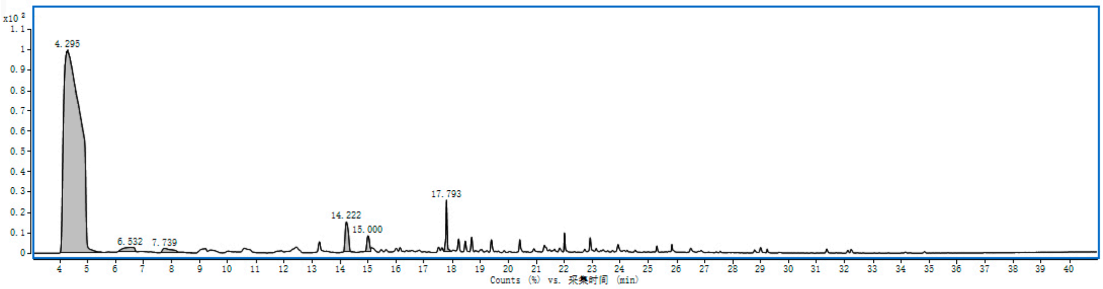

（f）

Figure S1. GC–MS total ion chromatograms of Ten-year aged Qian-he cellar vinegar (a), Ning-hua-mansion old vinegar (b), East-lake health vinegar (c), Qian-he glutinous rice vinegar (d), Heng-shun Jinyou balsamic vinegar (e), and potato vinegar (f)

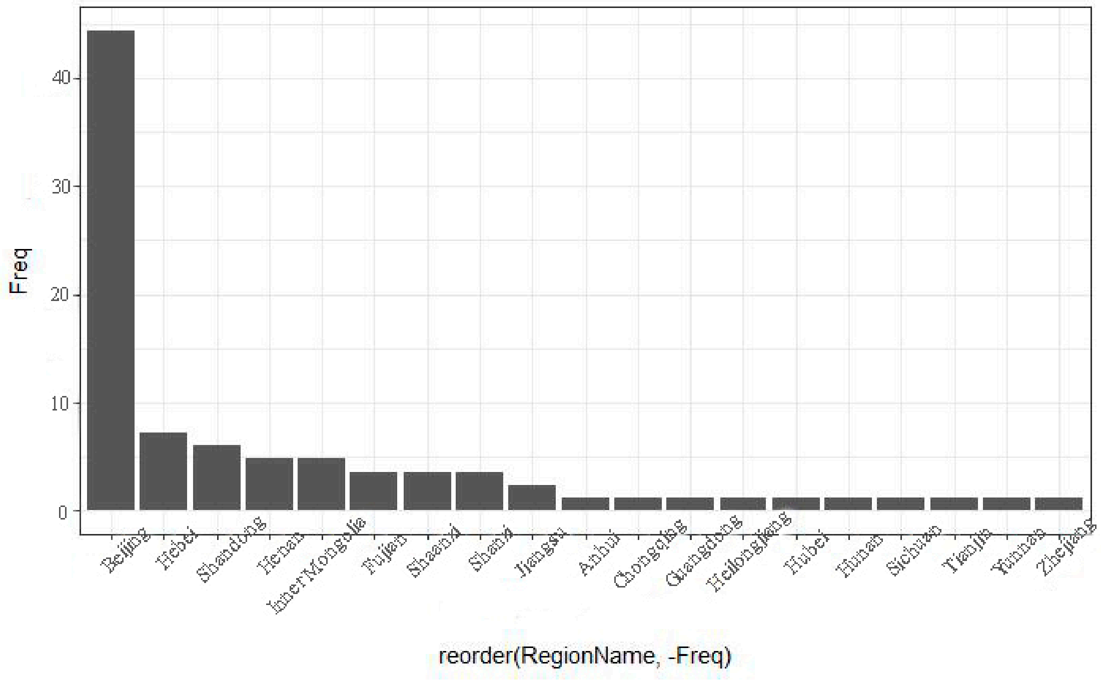

Figure S2. Geographical distribution of respondents

Table S1. Coefficient of PLSR models for each cluster of consumers

Compounds	Cluster 1	Cluster 2	Cluster 3
(Intercept)	3.456050575	2.900285975	4.191192809
ethyl acetate	0.052992195	0.041256449	-0.070823343
ethyl propionate	0.033226395	-0.038808468	0.021024787
n-propyl acetate	0.015631291	0.033368651	-0.041774873
isobutyl acetate	-0.066571488	0.02835015	0.042447526
isoamyl acetate	-0.06560561	0.041076224	0.031000854
1,2-propanediol,2-acetate	-0.009335841	-0.105296627	0.098242768
trimethylene acetate	0.019216632	0.024812648	-0.021426136
ethyl benzoate	-0.040300354	0.011777259	0.00356481
diethyl succinate	-0.000520397	-0.000989915	-0.027134602
β-phenethyl acetate	-0.029356264	0.011517687	-0.009063298
3-methylbutyraldehyde	-0.031357201	-0.019869663	0.023904848
benzaldehyde	0.001445659	0.105303412	-0.079789235
phenylethanal	-0.029101826	0.060336093	-0.019420937
1H-pyrrole-2-carbaldehyde	0.049832435	-0.032009312	-0.016141079
1-methylpyrrole-2-carboxaldehyde	0.056461276	-0.0558709	-0.00541892
acetic acid	0.030254114	-0.073879974	0.011811463
propionic acid	0.000872921	0.033833727	-0.014087127
butyric acid	0.009977118	-0.022896813	-0.018547984
isovaleric acid	-0.010305801	-0.0076494	-0.012206123
2-methylbutyric acid	-0.031056199	-0.038246816	0.034674256
caproic acid	0.02837145	-0.047412738	-0.010856694
octanoic acid	0.012979257	0.018098937	-0.020847446
3-methyl-1-butanol	-0.062073097	0.064716415	0.005063059
2,3-butanediol	0.023296506	-0.018329507	-0.017762306
phenethyl alcohol	-0.048565622	0.028894366	0.000362014
3-hydroxy-2-butanone	0.008852308	0.058896707	-0.040114937
acetophenone	0.024755389	-0.01075851	-0.012285165
2-pyrrolidinone	0.015366998	0.013310501	-0.02569417
guaiacol	0.03433283	-0.00821627	-0.019156161
4-ethyl-2-methoxyphenol	0.009974038	0.043634815	-0.033003297
4-ethylphenol	0.032232916	-0.004745407	-0.020376677
furfural	0.012935676	0.005873907	-0.029868392
acetylfuran	0.055803374	-0.03347207	-0.024573253
furfuryl acetate	-0.028128301	-0.012741882	0.029009408
1-pentanone, 1-(2-furanyl)-	0.030733207	-0.008019582	-0.015948382
3-furanmethanol	0.018710568	-0.045957467	0.020376849
1-(5-methyl-2-furyl)ethan-1-one	0.034481453	-0.008259147	-0.019263956
4-(2-furyl)-3-buten-2-one	0.04267412	-0.057801153	0.012151728
5-acetyldihydrofuran-2(3H)-one	0.014780012	0.012897748	-0.024650172

2,3-dimethyl pyrazine	0.04513335	-0.012656225	-0.02329515
2,3,5-trimethylpyrazine	0.045061374	-0.01499961	-0.019847758
1,3-dioxolane,2,4,5-trimethyl-	-0.016288298	0.05002548	-0.049529314
naphthalene	0.001916086	0.031807084	-0.013324674
2-methylnaphthalene	0.026804324	0.001872652	-0.013945558
2-phenylthiophene	0.014762988	-0.032287877	0.0133418
4-acetoxy-3-methoxystyrene	-0.029428267	-0.090655639	0.099931345

n = 30 for cluster 1, n = 18 for cluster 2 and n = 28 for cluster 3.
